# Green synthesis of silver nanoparticles from *Brassaiopsis hainla* extract for the evaluation of antibacterial and anticorrosion properties

**DOI:** 10.1016/j.heliyon.2024.e35642

**Published:** 2024-08-03

**Authors:** Sujan Budhathoki, Nabina Chaudhary, Biswash Guragain, Dipak Baral, Janak Adhikari, Narendra Kumar Chaudhary

**Affiliations:** a Department of Chemistry, Mahendra Morang Adarsh Multiple Campus, Biratnagar, Tribhuvan University, Nepal; b Department of Intensive Care Unit, Birat Medical College and Teaching Hospital, Biratnagar, Nepal

**Keywords:** Green synthesis, *Brassaiopsis hainla*, AgNPs, Antibacterial, anti-Corrosion

## Abstract

Plant-mediated synthesis of silver nanoparticles (AgNPs) is an eco-friendly and convenient alternative to conventional methods. *Brassaiopsis hainla (B. hainla)* leaf extract (BHE) was used in this study to reduce metal salts and cap and stabilize nanoparticles (NPs), which were characterized and tested for antibacterial and anti-corrosion properties. Stirring the *B. hainla* extract with AgNO_3_ led to a color change, indicating nanoparticle formation. The absorption peak at 428 nm in the UV–visible spectrum further validated its formation. The AgNPs were characterized using various techniques such as FTIR, UV–visible, PXRD, HRTEM, SEM, and EDX. Powder X-ray diffraction analysis confirmed its nanocrystalline nature, with an average crystallite size of 17.92 nm. The FTIR spectrum showed hydroxyl, amine, amide, and carbonyl groups as capping and reducing agents for the AgNPs. SEM analysis revealed poly-dispersed NPs of various sizes, while EDX showed an intense peak for Ag, and TEM images revealed mostly hexagonal and triangular NPs. Antibacterial activity was tested against three human pathogens: *Staphylococcus aureus (S. aureus), Pseudomonas, and Klebsiella oxytoca (K. oxytoca).* Significant antibacterial activity was observed specifically against *K. oxytoca*, with an 11 mm inhibition zone. Both plant extracts and AgNPs inhibited acid-induced corrosion, with the highest inhibition efficiencies of 81.69 % and 69.54 % at 1000 ppm, respectively. With rising concerns over bacterial resistance and metal corrosion, this study addresses global challenges related to new antimicrobial agents, which are crucial for combating antibiotic resistance and protecting metals in various industries.

## 1 Introduction

The use of plant extracts for the synthesis of nanoparticles (NPs) has gained significant attention in recent years because of their environmentally friendly nature and low toxicity [1,2]. It leverages the natural processes of plants to reorganize inorganic metal ions into nanoparticles, making them biocompatible with biological cells. Nanoparticles can then deliver targeted drugs and therapies to cells, thus improving efficacy and reducing adverse effects. A revolutionary change in the manner in which drugs are administered may be possible using this technology. Thus, green synthesis offers a cost-effective and biologically safe approach for producing nanoparticles with desired antibacterial and anticorrosive properties [3]. NPs exhibit different physical, chemical, and biological properties because of their high surface area-to-volume ratio, which increases their stability and reactivity in chemical processes [4,5]. They have been used in various fields of science, such as nanomedicine, catalysis, and drug delivery [6]. Emerging diseases require rapid action and potent drugs for effective treatment without adverse effects. With the global spread of drug resistance, antibiotics have become less effective, rendering infections more difficult to treat. Because metallic NPs have unique shapes and sizes, they have been applied in fields such as biomedicine, optics, catalysis, and photography [7,8]. AgNPs are widely used in medicine, cosmetics, agriculture, and household appliances because of their large surface area-to-volume ratio, chemical stability, and high thermal and electrical conductivity [9]. Their significant antimicrobial properties make them suitable for medical applications, including the inhibition of biofilm formation and reduction of pathogenic invasion [10].

**Fig. 1:**
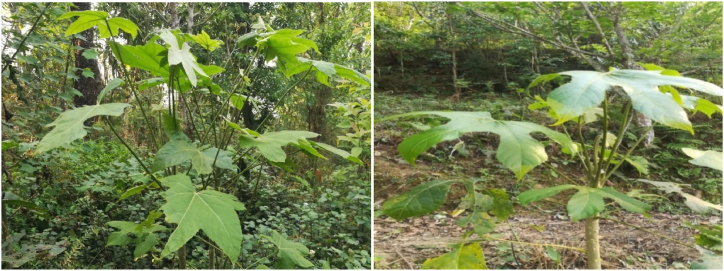
*Brassaiopsis hainla* plant.

*B. hainla* leaf extract contains high amounts of phytochemicals such as alkaloids, glycosides, flavonoids, steroids, saponins, and proteins (Fig. 1) that serve as reducing and capping agents for NP formation [11]. Therefore, they can be evaluated for their ability to combat bacterial infections and protect against corrosion. In one study, AgNPs synthesized from pigeon pea leaf extract exhibited strong anticorrosion properties [12], and those synthesized from *Lysiloma acapulcensis* extract exhibited significant antibacterial activity [13]. These studies emphasize the versatility and effectiveness of plant-mediated synthesis of AgNPs, prompting further exploration of other plant species, such as *B. hainla*. Further research is needed to explore the potential antibacterial and anticorrosion properties of the AgNPs synthesized from this plant. Furthermore, it is possible to assess the overall biocompatibility of these nanoparticles to ensure their safe use in various applications [14,15]. The low toxicity of this plant, combined with its phytochemical constituents, greatly contributes to its versatility. In contrast to conventional chemical and physical methods, plant extracts offer several advantages for NP synthesis. Additionally, plant extracts can provide more control over the surface properties of NPs, such as their charge, and stability [16,17]. Green synthesis using plant extracts eliminates the need for toxic chemicals, which can damage the environment and human health. Therefore, it is a safe and sustainable alternative for NP production [18].

**Fig. 2:**
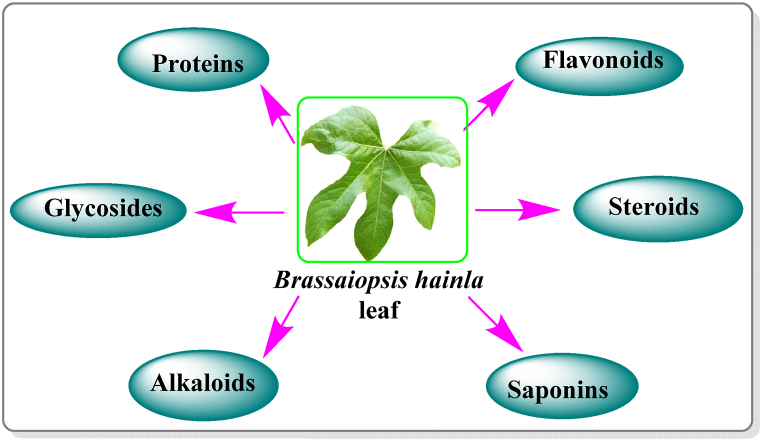
Phytochemicals present in *Brassaiopsis hainla* leaf extract.

*B. hainla*, an *Araliaceae* shrub (Fig. 2) known locally as chuletro, is found in the hilly regions of Nepal, including Illam and Dhankuta. It is a deciduous tree, widely distributed in Bhutan, Bangladesh, northern India, Myanmar, Thailand, and South-Central China. This species is found in the forests of the East Himalayas of Nepal between 1300 and 2100 m above sea level. The leaves contain 22 % crude protein, making them in high demand as animal fodders [19]. The current study explored the green synthesis of AgNPs using *B. hainla* leaf extract, which was characterized by UV–visible, FTIR, PXRD, SEM-EDX, and HR-TEM studies. In this study, we investigated the antibacterial activity of AgNPs against three different pathogenic bacteria: S*. aureus, Pseudomonas*, and *K. oxytoca*. Furthermore, the anti-corrosion profile was also investigated to highlight its importance.

## 2 Experimental

### 2.1 Materials and methods

The studied plant was collected from Rajarani in the Dhankuta district, Koshi province of Nepal (26°53′10.1″N, 87°25′32.6″E). It was identified and authenticated by the Department of Botany at the Mahendra Morang Adarsh Multiple Campus, Biratnagar. Silver nitrate from Qualigens (Thermo Fisher India Ltd.) was used to synthesize NPs. Muller Hinton's agar, nutrient broth, amikacin, and acetone were purchased from HiMedia Laboratories Pvt. Ltd. (India). Dimethyl sulfoxide (DMSO) was purchased from Thermo Fisher Scientific India Pvt. Ltd. Double-distilled water was used to clean and rinse glassware. All chemicals and reagents used were of AR grade, and no further purification was required.

### 2.2 Preparation of *B. hainla* extract

Fresh leaves of the plant were washed with tap water followed by distilled water to remove impurities, and then air-dried for approximately 20 days. The dried leaves were pulverized into fine powder using a mixer. The powder sample (10 g) was mixed with 100 mL of double-distilled water in a 250 mL RB flask, and heated to boiling for 50 min at 60 °C using a water condenser. The mixture was filtered through Whatman No. 1 filter paper, and the filtrate was stored at 4 °C for future use [20].

### 2.3 Synthesis of AgNPs

**Scheme 1:**
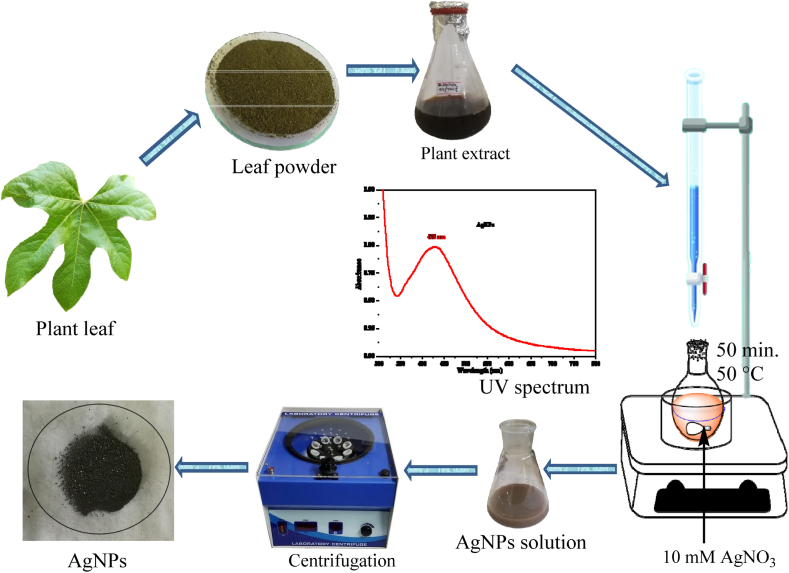
An outline of the synthesis of AgNPs.

**Fig. 3:**
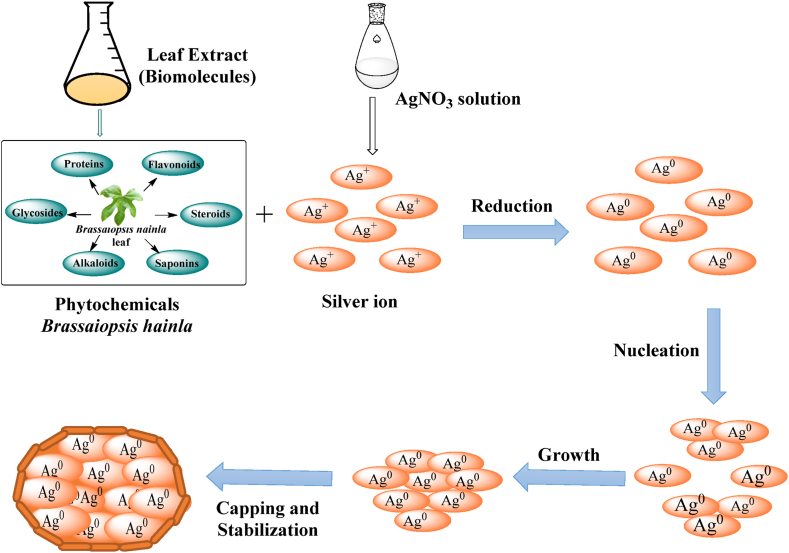
Synthesis mechanism of AgNPs from *B. Hainla* extract.

Optimal conditions, such as concentration, pH, temperature, reactant volume ratio, and reaction time, were adjusted to control the nanoparticle size, shape, and stability, making this method versatile and adaptable to different applications. Considering all experimentally optimized reaction conditions, AgNPs were synthesized according to a previously reported literature [21,22]. Twenty milliliters of *B. hainla* leaf extract was added dropwise to 80 mL of 10 mM AgNO_3_ solution and stirred at 60 °C by adjusting the reactant volume ratio (2:8) and pH to 9 in a 250 mL Erlenmeyer flask. Initially, the color was a light yellowish hue; however, over time, it gradually darkened and turned deep brown. After stirring for 1 h, the final color changed to gray, indicating completion of the NP formation reaction. The formation of NPs was detected using UV–visible spectroscopy. The flask was refrigerated for 24 h to ensure the stability of the NP. The solution was centrifuged at 3500 rpm for 25 min to separate and purify the AgNPs. Double-distilled water was used to wash it, followed by 99 % ethanol. The AgNP pellets were then dried for 48 h in an oven [23]. A schematic of the synthesis of AgNPs and the mechanism of AgNP formation from *B. hainla* leaf extract is shown in Scheme 1 and Fig. 3, respectively.

### 2.4 Characterization techniques

An initial analysis of the AgNPs was performed using UV/visible spectroscopy. This study analyzed the spatial information of groups linked to a sample of plant extracts and NPs. The functional groups responsible for reducing and stabilizing the NPs were identified using a PerkinElmer 783 FT-IR spectrometer in the range of 4000-400 cm^−1^. The X-ray diffractometer used was a vertical Philips 1130/00 model, operated at 40 kV and 50 MA generators, with monochromatic Cu-K_α_ radiation at a wavelength of 1.54056 Å. SEM images of the synthesized nanoparticles were obtained using a JEOL 6390LV/OXFORD XMX N instrument. The elemental composition was determined using energy-dispersive X-ray spectroscopy (EDX). HR-TEM analysis was performed using a JEOL/JEM 2100 transmission electron microscope (TEM).

### 2.5 Antibacterial activity study

The modified Kirby-Bauer paper disk diffusion technique was used to assess the antibacterial activity of the plant extract and AgNPs [24]. Fresh specimens of gram-positive (*S. aureus*) and gram-negative bacteria (*Pseudomonas* and *K. oxytoca*) were collected from the biochemistry laboratory of Koshi Zonal Hospital, Biratnagar, Nepal, and were revived in nutrient broth. All the glassware and solutions needed for this study were autoclaved to sterilize them. Approximately 20 mL of hot Mueller Hinton Agar (MHA) solution was poured onto Petri plates, and the solution was allowed to solidify for a while. The revived bacterial culture was carefully transferred to MHA media on Petri plates using a sterile stick swab. Circular paper discs loaded with 5 μL test solutions were placed on swabbed media, labeled, and incubated at 37 °C for 24 h. The diameter of the zone of inhibition was measured using an antibiogram zone measuring scale.

### 2.6 MIC and MBC study

The minimum inhibitory concentration (MIC) is the lowest concentration of a chemical that prevents visible growth of bacteria under specific conditions. A stock solution of AgNPs and plant extracts was prepared at concentrations of 12.5 g/L and 25 g/L and then further diluted using a broth microdilution technique to obtain lower concentrations. 25 μL of the bacterial suspension was added to the test solutions and incubated at 37 °C for 24 h to observe the highest dilution without turbidity [25]. The minimum bactericidal concentration (MBC) is the lowest concentration of a test sample required to eliminate 99.9 % of the test bacteria. A sterile Petri dish containing Mueller Hinton agar was streaked with bacterial samples, and the plates were incubated at 37 °C for 24 h. The minimum bactericidal concentration (MBC) was determined as the concentration at which no visible bacterial growth was observed.

### 2.7 Corrosion inhibition study

The plant extract and silver nanoparticles (AgNPs) were tested for their ability to inhibit carbon steel corrosion in HCl solution using the weight loss method. Dried plant extract (0.12 g) and AgNPs were separately dissolved in 120 mL of a 1 M HCl solution to prepare a 1000 ppm stock solution. The solutions were diluted to 800, 600, 400, and 200 ppm by adding 1 M HCl. A metal sheet was cut into 18 coupons of 2 cm × 2 cm size, and polished to a smooth surface using various grits of 60, 150, 320, 600, 800, 1000, 1500, and 2000 number emery (silicon carbide) papers. The steel coupons were then cleaned with distilled water and acetone. Their size and weight were measured using a digital screw gauge and a 4-digit digital balance. They were immersed in test solutions with varying concentrations of AgNPs and plant extracts at room temperature. One coupon immersed in 1 M HCl served as positive control. After 6 h of immersion, the samples were removed, washed, and weighed. The coupons were weighed in triplicate, and the average weight loss was used to calculate the corrosion rate, inhibition efficiency, and surface coverage [26] using the following equations:

(1) $$\text{Corrosion}\;\text{Rate}\;\left(\text{CR}\right)=\frac{87600\times \Delta w}{d\times A\times t}$$

(2) $$\text{Inhibition}\;\text{efficiency}\;\left(\eta \right)=\frac{CR-CR^{\prime }}{CR}\times 100\;\%$$

(3) $$\text{Surface}\;\text{Coverage}\;\left(\theta \right)=1\;-\frac{w_{2}}{w_{1}}$$

where "Δw" represents weight loss in grams; ‘d' represents the density of carbon steel in grams/cm^3^; ‘A' represents the area of carbon steel coupons in cm^2^; and ‘t' represents immersion time in hours. CR and CR’ are the corrosion rates in the absence and presence of inhibitor, respectively. w_1_ and w_2_ are the weight loss in the absence and presence of the inhibitor, respectively.

## 3 Results and discussion

### 3.1 UV-VIS spectral analysis

**Fig. 4:**
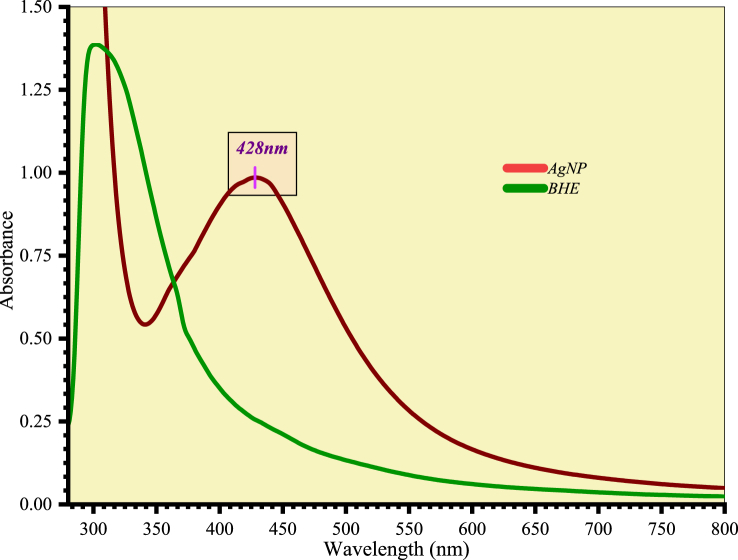
UV–visible spectrum of AgNPs and BHE.

The UV–visible spectrum of the reaction mixture is shown in Fig. 4. The maximum absorbance peak of AgNPs was observed at 428 nm, which is very close to the reported value [27]. The broad peak observed in the range of 400–450 nm was attributed to the surface plasmon resonance (SPR) of AgNPs [28,29]. SPR is caused by the oscillation of electrons in the solution in response to light [30]. The SPR peak displaying the photogenic characteristics of AgNPs was analyzed by observing the alteration in color. The brown color of the reaction mixture indicated that Ag^+^ was reduced to Ag^0^, confirming the formation of AgNPs [31,32]. The broadening of the peak and absorption bands above 420 nm indicated poly-dispersed particles, which was confirmed by HR-TEM analysis [33]. The shape, size, and surrounding environment of metallic NPs affect the resonance frequency of surface plasmons [34].

### 3.2 FTIR spectral analysis

**Fig. 5:**
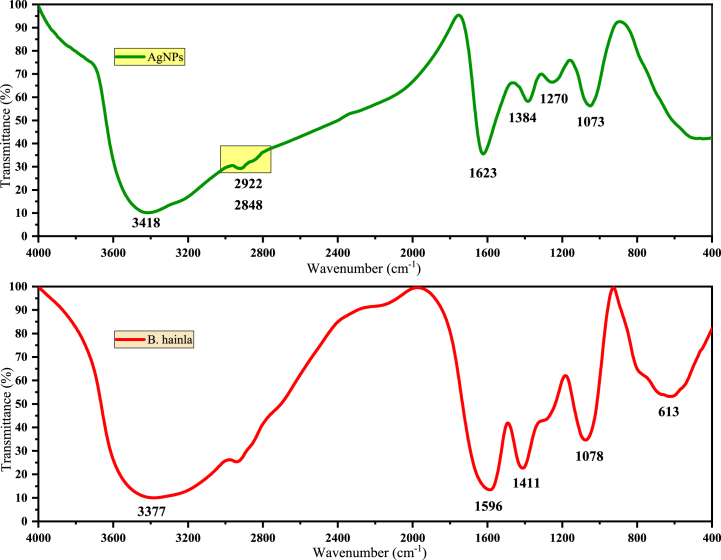
FTIR spectrum of AgNPs and BHE.

FT-IR spectroscopy was performed to identify the functional moieties responsible for stabilizing and reducing Ag^+^ ions and capping bio-reduced AgNPs (Fig. 5). The broad absorption peak with maximum absorbance at 3377 cm^−1^ for the plant extract may be due to –OH, and –NH stretching vibrations, which shift to 3418 cm^−1^ in the spectrum of AgNPs [35]. The less intense peaks at 2922 cm^−1^ and 2848 cm^−1^ were attributed to methyl (C–H) stretching vibrations [36]. The absorption band observed at 613 cm^−1^ in the spectrum of AgNPs was attributed to the C≡C bending vibration, and some of the bands disappeared after NP formation [37]. Moreover, the more intense peaks observed at 1623 cm^−1^ and 1270 cm^−1^ in the spectrum of AgNPs confirmed the involvement of functional groups in the bio-reduction mechanism leading to AgNP formation. The spectra also show a significant shift associated with the amide (1651–1630 cm^−1^) linkage, suggesting that the amino (-NH_2_) and carboxylate (-COO^-^) groups in the plant extracts interact with AgNP surfaces. This interaction results in the greater stability of AgNPs [38]. The peak corresponding to the C–N stretch of the amine group at 1411 cm^−1^ was broad and became confined and sharper at 1384 cm^−1^ after nanoparticle encapsulation. The C–O stretching vibration peak for the plant extract at 1078 cm^−1^ shifted to 1073 cm^−1^ in the spectrum of AgNPs, suggesting stabilization by this group [39]. Overall, FTIR studies indicated that phytochemicals bind metals and form a protective layer over metal nanoparticles (e.g., capping AgNPs) to prevent their agglomeration and stabilize them [40].

### 3.3 SEM-EDX analysis

**Fig. 6:**
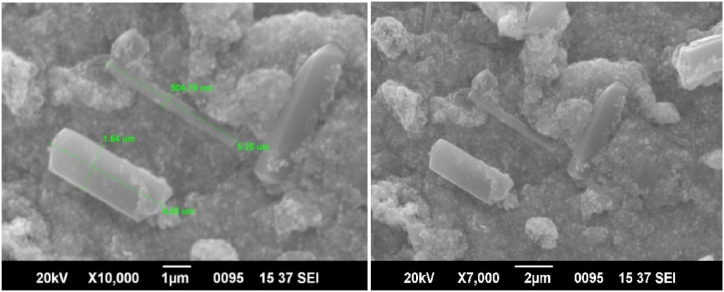
SEM images of AgNPs.

Scanning electron microscopy (SEM) was performed to observe the topology and size of the NPs. SEM micrographs of AgNPs (Fig. 6) show variously sized polydisperse nanoparticles. They displayed large agglomerated particles, formed by overlapping of smaller particles [41]. AgNP clusters may have formed because of nanoparticle aggregation during the sample preparation [42]. Several particles exhibit hexagonal shapes with varying diameters.

**Fig. 7:**
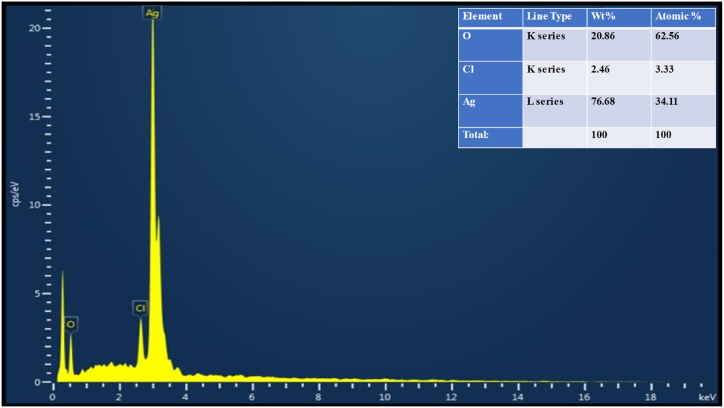
EDX image of AgNPs.

The EDX analysis validated the elemental composition of the synthesized AgNPs (Fig. 7). Based on EDX data, Ag accounted for 76 % of the weight, while O and Cl elements accounted for 20.86 % and 2.46 %, respectively [43]. Spectral signals for oxygen and chlorine indicated that extracellular organic molecules were adsorbed onto the surface of nanoparticles [44]. AgNPs are generally stabilized in aqueous plant extracts by a thin layer of chain-ceasing compounds [45].

### 3.4 HR-TEM analysis

**Fig. 8:**
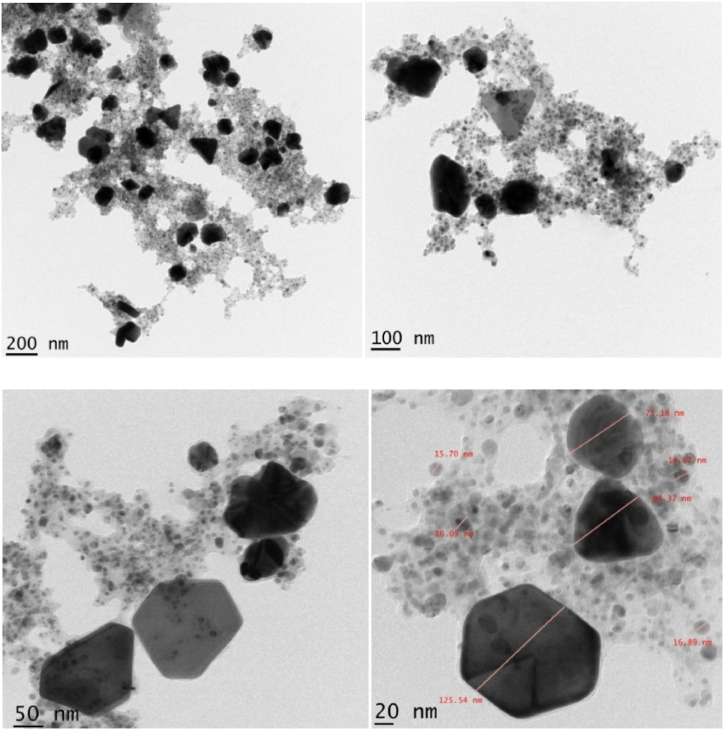
HRTEM micrographs of AgNPs.

**Fig. 9:**
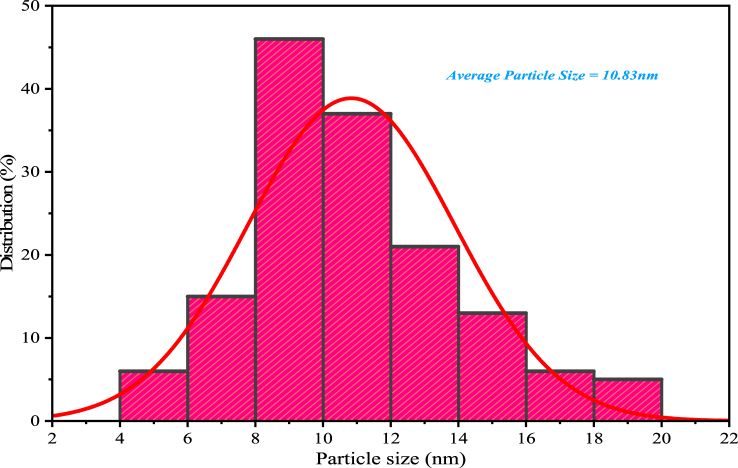
Histogram of particle size distribution of AgNPs.

High-resolution transmission electron microscopy provides information about the shape, morphology, size, dispersion, and crystallinity of the AgNPs. It can also be used to investigate the interaction of AgNPs with various materials, such as cells, proteins, and other molecules. This information is essential to understand the effects of AgNPs on human health and the environment. Fig. 8, Fig. 9 show HR-TEM images and a histogram showing the particle size distribution of the AgNPs. This study found their average size was 10.83 nm, and they had irregular shapes and sizes ranging from 2 to 125 nm. Based on the TEM micrographs, the nanoparticles predominantly had hexagonal and triangular shapes [46].

### 3.5 PXRD analysis

**Fig. 10:**
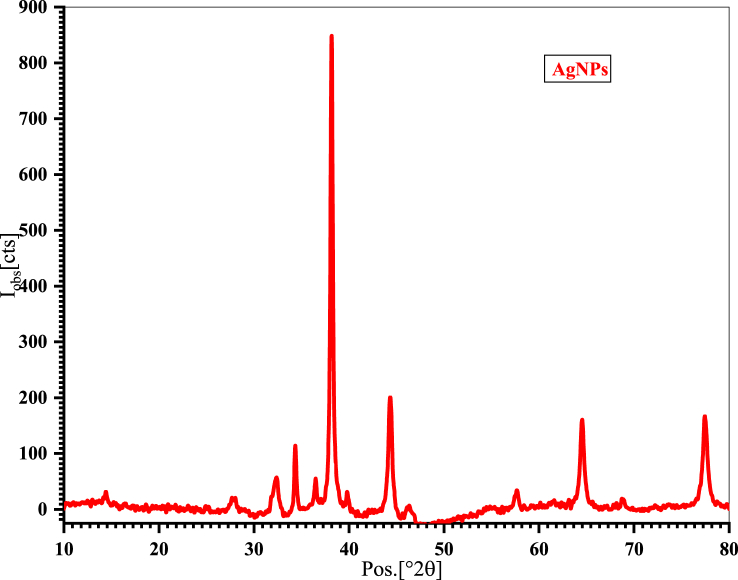
PXRD pattern of AgNPs.

PXRD was used to investigate the crystal properties of the nanoparticles, including crystallinity, crystallite size, dislocation density, and microstrain. Fig. 10 shows the diffractograms of the AgNPs. It displayed eight distinct peaks of varying intensities, with a maximum intensity peak at 38.17° and a corresponding d-spacing value of 2.356 Å. Unassigned peaks may result from the crystallization of the bioorganic phase on the surface of the NPs and were not considered in the calculation [47,48].

The average particle size was estimated according to the Debye–Scherrer formula.

$$D=\frac{K\lambda }{\beta \;\mathrm{Cos}\;\theta }$$

where, λ is the wavelength, β (radian) is the full width at half maxima (FWHM), and θ (radian) is the diffraction angle. K is a Scherrer constant called the shape factor [49]. The crystallite size was calculated for each of the eight peaks, and the average crystallite size was found to be 17.14 nm. The other parameters as including % crystallinity, crystallite size, dislocation density, and microstrain are presented in Table 1.

**Table 1:** Diffraction data of AgNPs.

No.	Pos. [°2θ]	d-spacing [Å]	FWHM [°2θ]	Crystallite size (D) (nm)	Dislocation density (x 10^−3)^	Microstrain (x10^−3)^
1	32.28	2.77	0.7483	10.92	8.37	9.45
2	34.36	2.60	0.3678	22.35	2.001	4.96
3	38.17	2.35	0.4149	20.03	2.49	6.26
4	44.33	2.04	0.6244	13.58	5.41	11.1
5	57.63	1.59	0.3724	24.07	1.72	8.94
6	64.53	1.44	0.5619	16.53	3.65	15.48
7	68.80	1.36	0.1777	53.57	0.34	5.31
8	77.47	1.23	0.6071	16.58	3.63	21.25
Average crystallite size (D_av_) = 17.92 nm						
Crystallinity = 45.11 %						

### 3.6 Antibacterial activity

**Fig. 11:**
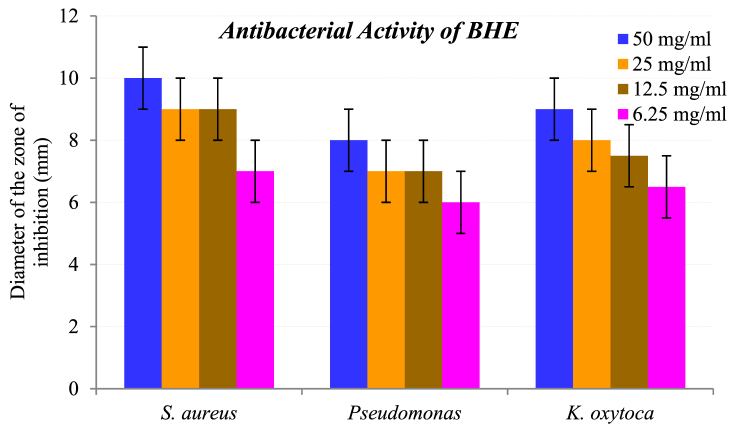
Antibacterial activity of BHE.

**Fig. 12:**
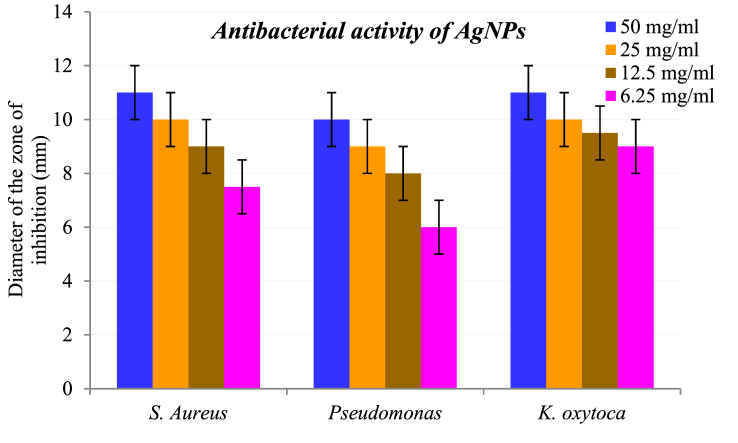
Antibacterial activity of AgNPs.

Table 2 shows the antibacterial inhibition data of *B. hainla* extract (BHE) and AgNPs. The data inferred a direct relationship between the inhibition efficiency and concentration of the tested compounds. Both the plant extracts and AgNPs displayed selective antibacterial activity against gram-positive and gram-negative bacteria. AgNPs showed a slightly better inhibitory effect than the plant extract after 24 h of incubation. The data analysis can be effectively visualized with the help of the bar graphs in Fig. 11, Fig. 12.

**Table 2:** Antibacterial activity data of BHE and AgNPs.

S. N.	Concentration (mg/ml)	*S. Aureus*		*Pseudomonas*		*K. Oxytoca*	
		BHE (mm)	AgNPs (mm)	BHE (mm)	AgNPs (mm)	BHE (mm)	AgNPs (mm)
1	50	10	11	8	10	9	11
2	25	9	10	7	9	8	10
3	12.5	9	9	7	8	7.5	9.5
4	6.25	7	7.5	6	6	6.5	9

BHE = *B. hainla* extract.

The highest inhibition zone was observed against the gram-negative bacterium *K. oxytoca* (11 mm) and the lowest was observed against *Pseudomonas* (10 mm) at a concentration of 50 mg/mL. Likewise, the *B. hainla* leaf extract exhibited greater activity against *S. aureus* (10 mm), and the least activity against *Pseudomonas* (8 mm). A difference in the membrane structure is primarily responsible for the higher resistance of gram-positive strains compared with gram-negative strains [50]. The dissimilarity in the results could be explained by variations in the interactions of AgNPs with the tested organisms and their susceptibilities. The enhanced antibacterial activity of AgNPs may be due to their small size and functional capping groups, which allow better interactions with bacterial membranes [51,52]. AgNPs are known to cause oxidative stress in bacteria by producing reactive oxygen species (ROS). ROS damage proteins, RNA, and DNA by attacking lipids in the cell membrane, causing lipid oxidation [53]. Likewise, Ag^+^ interacts with DNA to inhibit bacterial reproduction [27]. Although the mechanism of Ag^+^ ions for antibacterial effect is not well understood, it may be due to the electrostatic attraction between the positive charges of NPs and the negative charges of bacterial cell membranes [54].

The actual and precise antibacterial activities of the studied compounds were evaluated based on their MIC and MBC values. The data are presented in Table 3. The MIC and MBC values for AgNPs against *Pseudomonas* and *K. oxytoca* did not show any marked differences and with values of 0.223 and 0.446 mg/mL, respectively. For *S. aureus*, MIC and MBC values were 0.446 and 0.893 mg/mL, respectively. The values for *B. hainla* extract against *S. aureus* and *K. oxytoca* were 1.562 and 3.125 mg/mL, respectively. *Pseudomonas* showed slightly lower resistance than the others.

**Table 3:** MIC/MBC data of BHE and AgNPs.

Sample	*S. Aureus*		*Pseudomonas*		*K. Oxytoca*	
	MIC (mg/ml)	MBC (mg/ml)	MIC (mg/ml)	MBC (mg/ml)	MIC (mg/ml)	MBC (mg/ml)
AgNPs	0.446	0.893	0.223	0.446	0.223	0.446
BHE	1.562	3.125	6.25	12.5	1.562	3.125

### 3.7 Corrosion inhibition study

Table 4 presents data on corrosion rate, inhibition efficiency, and surface coverage for *B. hainla* leaf extract and AgNPs. Both samples inhibited acid-induced corrosion of the steel specimens compared with the blank solution. At the highest concentration, the *B. hainla* extract showed a better inhibition efficiency of 81.69 % compared to AgNPs (69.54 %). The extent of protection against corrosion in both samples is entirely reliant on the concentration of the samples. The inhibition efficiency increased with the rise in concentrations of both the plant extract and AgNPs.

**Table 4:** Corrosion parameters for BHE/AgNPs.

Sample	Concentration (ppm)	Corrosion rate (mm/year)	Inhibition efficiency (η) (%)	Surface coverage (θ)
**Control**	0	18.01	–	–
***B. Hainla*** **Extract**	200	6.19	65.15	0.6632
	400	5.08	70.78	0.7080
	600	4.56	73.09	0.7399
	800	3.88	77.23	0.7761
	1000	3.11	81.69	0.8145
**Control**	0	16.99	–	–
**AgNPs**	200	8.22	51.38	0.5121
	400	6.99	58.62	0.5720
	600	6.30	62.98	0.6506
	800	5.43	67.91	0.6889
	1000	5.15	69.54	0.7181

**Fig. 13:**
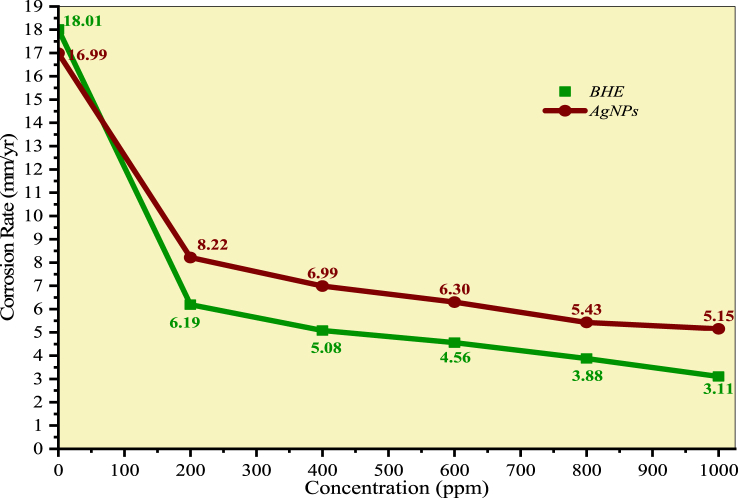
Corrosion rate of mild steel in BHE and AgNPs solution.

**Fig. 14:**
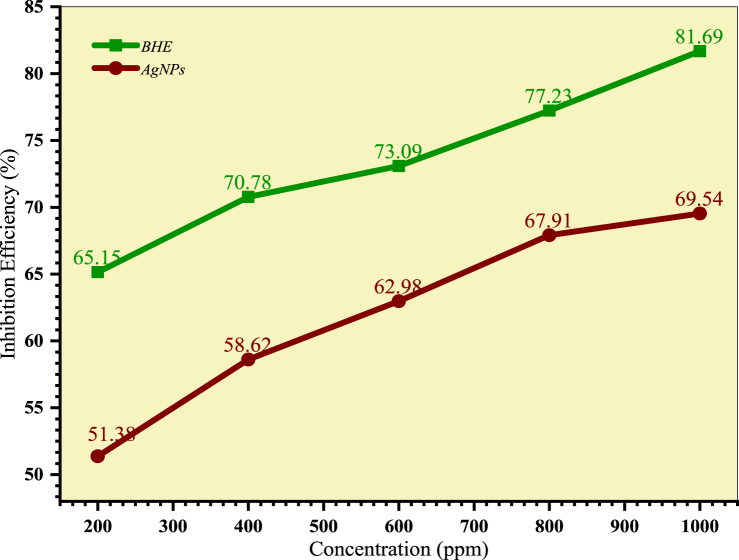
Inhibition efficiency of the BHE and AgNPs at various concentrations.

There is no clear reason for the lower corrosion inhibition efficiency of AgNPs compared to that of the plant extract, which might be due to its incomplete solubility in 1M HCl. This observation can be attributed to the presence of inhibitor molecules in the corrosive medium, which promote their adsorption on the metal surface [55]. The availability of inhibitor molecules of AgNPs in the corrosion medium might be limited, which could result in differences in the inhibition. Plant extracts contain various phytochemicals, such as tannins and phenols, which possess active adsorption sites, and interact with the vacant orbitals of iron through adsorption [56]. Fig. 13, Fig. 14 show the corrosion rate and inhibition efficiency of the plant extract and AgNPs, respectively.

### 3.8 Adsorption isotherm study

**Fig. 15:**
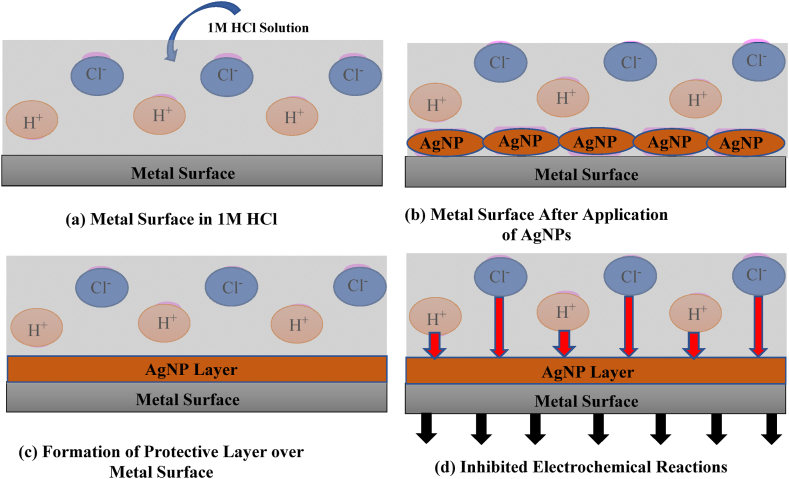
Schematic diagram showing the anti-corrosion mechanism of AgNPs: (a) Metal surface exposed to 1M HCl solution; Presence of corrosive agents (H⁺ and Cl⁻ ions) (b) Application of AgNPs (c) Formation of a protective AgNPs layer on the metal (d) The AgNPs layer acts as a physical barrier, blocking H⁺ and Cl⁻ ions from reaching the metal surface.

Generally, corrosion inhibitors block active sites by adsorption on the surface of the metal. Steel coupons immersed in 1M HCl without an inhibitor initially absorbed acid molecules, resulting in corrosion. In an acidic environment, inhibitor molecules readily adsorb onto the steel surface, effectively block reaction sites, and provide high protection against acidic ion attack [57]. A schematic diagram showing the anti-corrosion mechanism of AgNPs is depicted in Fig. 15(a–d). For a better understanding of the adsorption process, adsorption isotherms provide salient details of the interaction between the steel and inhibitor molecules, which may occur by the action of physical or chemical forces. A straight relationship between the inhibition efficiency and surface coverage was plotted for adsorption isotherms, such as Langmuir, to describe the nature of inhibitor adsorption on steel. Based on this model, the surface coverage (θ) is associated with its concentration as follows:

$$\frac{C_{inh}}{\theta }=\frac{1}{K_{ads}}+C_{inh}$$

where *C*_*inh*_ represents the inhibitor concentration (g/L) and *K*_*ads*_ indicates an equilibrium constant known as the adsorption coefficient [58].

**Fig. 16:**
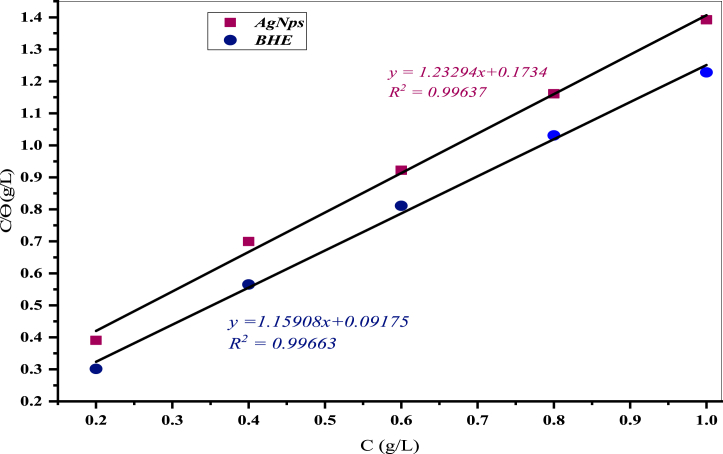
Langmuir adsorption isotherm plot with different concentrations of BHE and AgNPs.

A straight line was obtained by plotting $\frac{C_{inh}}{\theta }$ vs *C*_*inh*_, whose intercept value provides a quantitative evaluation of the adsorption constant (*K*_*ads*_). The fitting of the experimental data points yielded a correlation coefficient (R^2^) of 0.996 with slopes of 1.159 for BHE and 1.232 for AgNPs, confirming the applicability of the Langmuir adsorption isotherm. This is illustrated in Fig. 16. According to the results, the inhibitor molecules exhibit monolayer adsorption on the steel surfaces without any interaction between them. The adsorption constant (*K*_*ads*_)) was determined from the intercept of the straight line and used to calculate the free energy of adsorption (ΔG^0^) [59].

$${\Delta G}^{0}=-RTIn\left(1000K_{ads}\right)$$

where R is the universal gas constant, T is the absolute temperature, and 1000 is the concentration of water molecules in g/L. The adsorption constants of AgNPs and BHE were 0.811 and 0.8628, respectively. The free energy of adsorption (ΔG^0^) for both AgNPs and BHE are negative i.e., −16.60 kJ/mol and −16.65 kJ/mol respectively, indicating spontaneous and stable adsorption on the steel surfaces. Generally, absolute free energy is used to label adsorption mechanisms, such as chemisorption, physisorption, or a combination of both. A ΔG^0^ value less than or equal to −20 kJ/mol suggests a physisorption mechanism, whereas a value of approximately −40 kJ/mol or greater denotes chemisorption [60]. This study demonstrated that the adsorption of AgNPs and BHE inhibitors on steel in 1M HCl develops a physisorption mechanism.

## 4 Conclusion

Biosynthesis of AgNPs was successfully achieved using *B. hainla* leaf extract through a green synthesis approach. Because this synthetic method is cost-effective, eco-friendly, and sustainable, it demonstrates the potential of plant extracts as renewable sources for the bio-reduction of heavy metal ions. The efficiency of this method is highlighted by the rapid bio-reduction achieved through a direct one-step reaction. The formation of nanoparticles was monitored by color change and UV–visible spectroscopy. They were further characterized using FTIR, PXRD, SEM-EDX, and HRTEM studies. The biosynthesized AgNPs exhibited profound antibacterial activity, demonstrating their significance in biological applications. This research has been extended to further explore the anti-corrosion properties of AgNPs. This study is the first to demonstrate the significance of AgNPs synthesized from *B. hainla* leaf extract, thus providing a valuable contribution to green nanotechnology. These findings open the way for further investigation into the various uses of biogenic AgNPs and emphasize the importance of sustainable production methods.

## Funding

This study has no funding.

## Data availability statement

All the data are included in the manuscript and are available for the readers.

## CRediT authorship contribution statement

**Sujan Budhathoki:** Writing – original draft, Methodology, Investigation, Formal analysis, Data curation. **Nabina Chaudhary:** Writing – review & editing, Investigation. **Biswash Guragain:** Writing – review & editing, Methodology, Investigation. **Dipak Baral:** Writing – review & editing. **Janak Adhikari:** Formal analysis, Data curation. **Narendra Kumar Chaudhary:** Writing – review & editing, Validation, Supervision, Conceptualization.

## Declaration of competing interest

The authors declare that they have no known competing financial interests or personal relationships that could have appeared to influence the work reported in this paper.
